# Multiple similarly effective solutions exist for biomedical feature selection and classification problems

**DOI:** 10.1038/s41598-017-13184-8

**Published:** 2017-10-09

**Authors:** Jiamei Liu, Cheng Xu, Weifeng Yang, Yayun Shu, Weiwei Zheng, Fengfeng Zhou

**Affiliations:** 1 College of Software, Jilin University, Changchun, Jilin, 130012 China; 2 College of Computer Science and Technology, and Key Laboratory of Symbolic Computation and Knowledge Engineering of Ministry of Education, Jilin University, Changchun, Jilin, 130012 China

**Keywords:** Computational biology and bioinformatics, Biomarkers

## Abstract

Binary classification is a widely employed problem to facilitate the decisions on various biomedical big data questions, such as clinical drug trials between treated participants and controls, and genome-wide association studies (GWASs) between participants with or without a phenotype. A machine learning model is trained for this purpose by optimizing the power of discriminating samples from two groups. However, most of the classification algorithms tend to generate one locally optimal solution according to the input dataset and the mathematical presumptions of the dataset. Here we demonstrated from the aspects of both disease classification and feature selection that multiple different solutions may have similar classification performances. So the existing machine learning algorithms may have ignored a horde of fishes by catching only a good one. Since most of the existing machine learning algorithms generate a solution by optimizing a mathematical goal, it may be essential for understanding the biological mechanisms for the investigated classification question, by considering both the generated solution and the ignored ones.

## Introduction

This study focuses on the binary classification problem in the biomedical big data. A binary classification algorithm tries to tune the parameters of a machine learning model by optimizing the discrimination power of samples from two groups^1,2^. This problem setting is widely employed to facilitate the clinical drug trials between treated participants and controls^3^, the genome-wide association studies (GWASs) between participants with or without a phenotype^4,5^, and the biomarker screening procedures between patients of a specific disease and healthy controls of similar baseline characteristics^6^, *etc*.

Most of the biomedical classification algorithms are deterministic computational algorithms, and the same single solution will be generated for the same input dataset^7,8^. Modern biotechnologies may produce thousands or millions of data points, or features, for a single sample^9,10^, and this renders finding the globally optimal solution impossible within a reasonable period of time^9^. So almost all the existing biomedical classification algorithms are approximate algorithms and can only deliver locally optimal solutions^11,12^. For example, a Support Vector Machine (SVM) builds a hyperplane that maximizes the margin to two groups of samples^13^, and its classification performance heavily depends on the kernel function^14^. Even a special case of an SVM was proven to be NP-hard, so the general SVM model is also NP-hard^15^. A Naïve Bayes (NBayes) classifier is not NP-hard, but has a strong assumption the independence between features^16^. Feature selection may improve the performance of the classification algorithms by reducing the feature dimensions^17,18^.

A few challenges still remain to be resolved for biomedical classification problems. Firstly, it’s difficult to select the best solution from a few alternative ones with minor or even no performance differences. But most of the existing classification algorithms output only one solution, and discard all the other candidate solutions with similar classification performances, generated during the optimization procedure. Secondly, a classification algorithm usually has some strong assumptions on the dataset, and tries to optimize a mathematical function based on these assumptions. The complexity of biomedical big data does not necessarily follow these assumptions, *e.g*. SVM’s kernel function^14^ and Naïve Bayes’s inter-feature independence^16^. So even the mathematically global optimum of a classification algorithm may not be a biologically best solution.

So this study proposes the hypothesis that the current machine learning algorithms might have ignored a forest of similarly good trees by taking one of these trees as the final solution. We support this hypothesis with multiple similarly good solutions for two binary classification problems, *i.e*. disease diagnosis and biomarker detection. The existence of such similarly-well solutions suggests that picking one of them may not be a good choice, and the algorithm users may want to make the choices based on their own domain-specific expertises.

## Results and Discussion

### Multiple ELMs performed similarly well

We detected more than one ELM models with similarly good classification accuracies for both rounds of experiments, *i.e. Top20Features* and *AllFeatures*. Firstly, our ELM models outperformed all the models on the three datasets CNS, Adeno and DLBCL generated in^9^, and the maximum improvement 15.7% in accuracy was achieved on the dataset CNS. The ELM models performed slightly worse (0.4% in accuracy) than the best model generated by the feature selection algorithm CFS in^9^. But CFS recommended 56 features, compared with the 20 features by the Top20Features ELM models.

Table 1 also shows the existences of more than one solution with similarly well classification performances. For the two easy datasets Adeno and DLBCL, hundreds or even more of ELM models with very good classification performances exist. For the difficult dataset CNS, even the worst ELM model performs better than the best model reported previously^9^. And there exist three ELM models with the same classification accuracy 83.3% for both the models of Top20Features and AllFeatures on the dataset ALL2.

**Table 1:** Summary of the best ELM models trained on the four datasets.

Dataset	ALL2	CNS	Adeno	DLBCL
ELMs in *Top20Features*	3	1673	10000	859
ELMs in *AllFeatures*	3	313	9994	174
MinAcc(McTwo)	0.651	0.643	0.878	0.914
MaxAcc(McTwo)	0.837	0.843	0.919	0.987
Top20Features: MinAcc(ELM)	0.833	0.944	1.000	1.000
Top20Features: MaxAcc(ELM)	0.833	1.000	1.000	1.000
AllFeatures: MinAcc(ELM)	0.833	0.889	1.000	0.958
AllFeatures: MaxAcc(ELM)	0.833	1.000	1.000	1.000

The models with accuracies larger than 0.800 were collected for the two difficult datasets ALL2 and CNS, and the accuracy cutoff 0.900 was used for the two easy datasets Adeno and DLBCL. Except the heading row, the first two rows are the number of ELM models using the training matrices Top20Features and AllFeatures, respectively. The next two rows “MinAcc(McTwo)” and “MaxAcc(McTwo)” gave the minimum and maximum binary classification accuracies of the models generated on the same datasets^9^. The minimum and maximum accuracies of the ELM models with the accuracies larger than the cutoff were listed in the last four rows.

Support Vector Machine (SVM) and Naïve Bayes (NBayes) are two widely used classifiers, and were also evaluated on the *T20Features* experiment of the two difficult datasets ALL2 and CNS. They are deterministic classification algorithms, and will generate the same models if the input datasets do not change. When the default random seed 0 was used, neither SVM and NBayes achieved accuracies better than 0.8. So this study conducted another randomized experiments for SVM and NBayes. The dataset was randomly split into 70% training and 30% testing datasets, respectively. An accuracy was calculated for the testing dataset using the model trained over the training dataset. 10,000 random runs were carried out, but no SVM or NBayes models achieved accuracies better than 0.8 on the difficult dataset ALL2. For the other difficult dataset CNS, there were 471 SVM models with accuracies better than 0.8, and NBayes established 1,874 models with accuracies better than 0.8. So in summary, ELM outperformed SVM and NBayes on the two difficult datasets, and may be a good candidate algorithm to investigate the hypothesis in this study.

**Figure 1:**
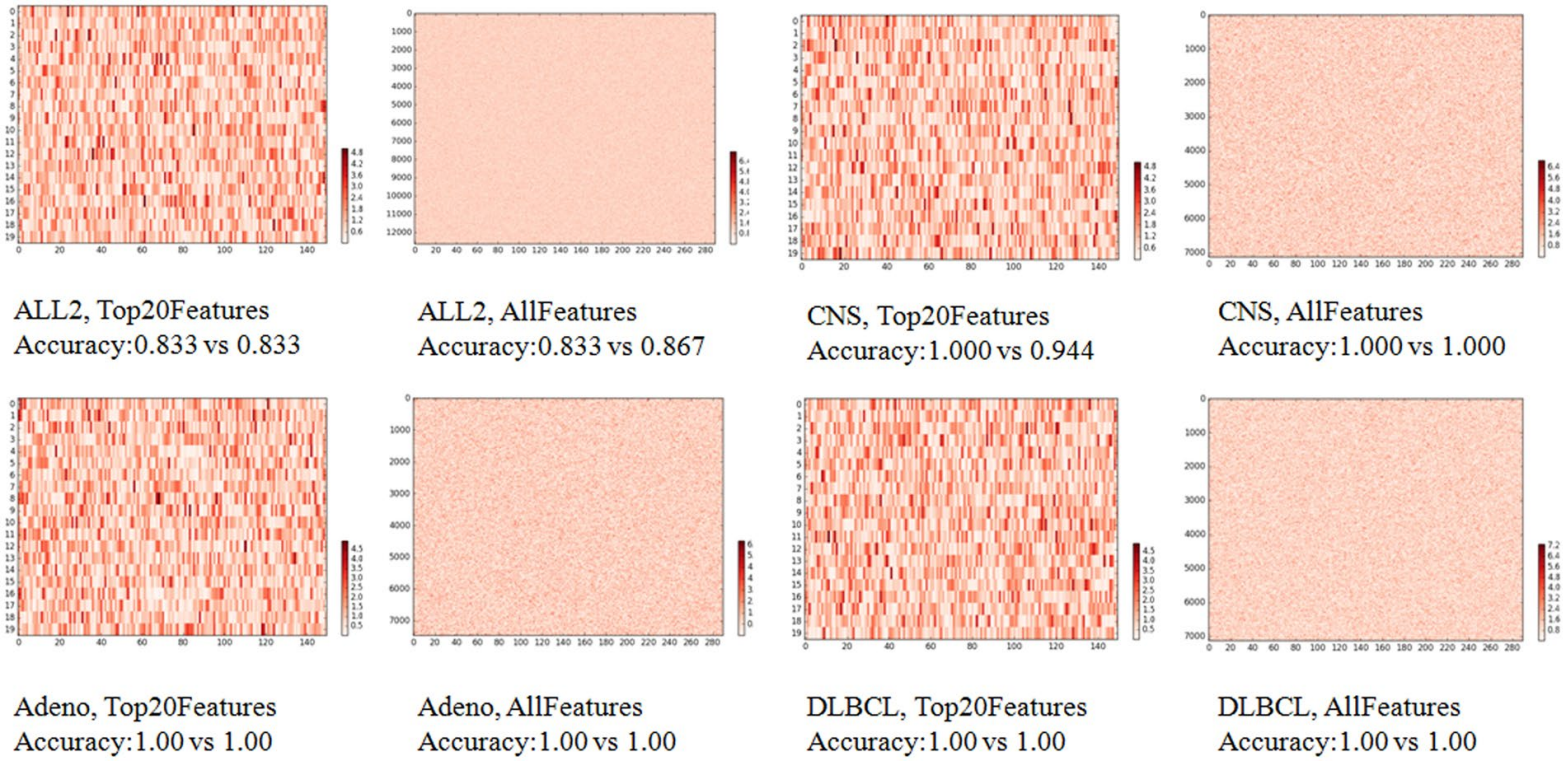
A heatmap of the difference matrix between the best two ELM models for the four datasets. If more than two ELM models achieve the best classification accuracy, two models were randomly chosen from these best ones.

Some of these accurate ELM models are significantly different to each other. An ELM model was represented by the matrix of the internal parameters, as described above. A heatmap was generated for the difference matrix between two ELM models, as shown in Fig. 1. A white color represents no difference between the corresponding weights in the two ELM models, and red represents the maximum difference. For the two best ELM models on the dataset ALL2, the maximum difference reaches 4.8 for the mode Top20Features, and a larger difference 6.4 for the model AllFeatures. A significant difference was observed in both heatmaps on the dataset ALL2. Similar patterns were observed for the other three datasets, as illustrated in Fig. 1.

### There exist multiple feature selection solutions with best classification accuracies

Firstly, more than one feature selection solution achieved very good performances, as demonstrated in Table 2. The best feature evaluated by the individual t-test didn’t always appear in the good solutions of all the four datasets, and even the features with ranks larger than 30 may work very well together with the other features, as in the case for the dataset ALL2 in Table 2. Even the worst triplet achieved the overall accuracy 0.830 for the dataset ALL2, which was similar to the best performance 0.837 in the previous study^9^. And the best feature triplet outperformed the best previous model by 0.030 in the overall accuracy. Table 2 showed that there are 1,707 triplets achieved accuracy larger than or equal to 0.80 for the dataset CNS, and even the worst feature triplet outperformed the previous best model by 0.101 in the overall accuracy. There are 19,040 and 6,803 feature triplets achieved 1.000 in accuracy for the two easy datasets Adeno and DLBCL, respectively.

**Table 2:** Summary of the best ELM models trained on triplets of the four datasets.

Dataset	ALL2	CNS	Adeno	DLBCL
Triplets	21	1707	19040	6803
MinAcc(McTwo)	0.651	0.643	0.878	0.914
MaxAcc(McTwo)	0.837	0.843	0.919	0.987
MinAcc(ELM)	0.830	0.944	1.000	1.000
MaxAcc(ELM)	0.867	1.000	1.000	1.000
(Min, Max) ranks	7.000, 32.000	4.000, 36.333	1.333, 29.000	12.667, 32.333

The models with accuracies larger than 0.800 were collected for the two difficult datasets ALL2 and CNS, and the accuracy cutoff 0.900 was used for the two easy datasets Adeno and DLBCL. Except the heading row, the first row is the number of ELM models using the training matrices with 3 features. The next two rows “MinAcc(McTwo)” and “MaxAcc(McTwo)” gave the minimum and maximum binary classification accuracies of the models generated on the same datasets^9^. The minimum and maximum accuracies of the best ten ELM models with the accuracies larger than the cutoff were listed in the following two rows. And the last row shows the averaged rankings of the 10 triplets with the best accuracies.

The averaged ranks of the selected triplets demonstrated the existences of many features that were not high in ranks but constituted very good feature triplets. The minimum feature ranks of these triplets were larger than 1.000 for all the four datasets. So there exist triplets that didn’t consist of the feature with the smallest t-test Pvalue for all the four datasets.

**Figure 2:**
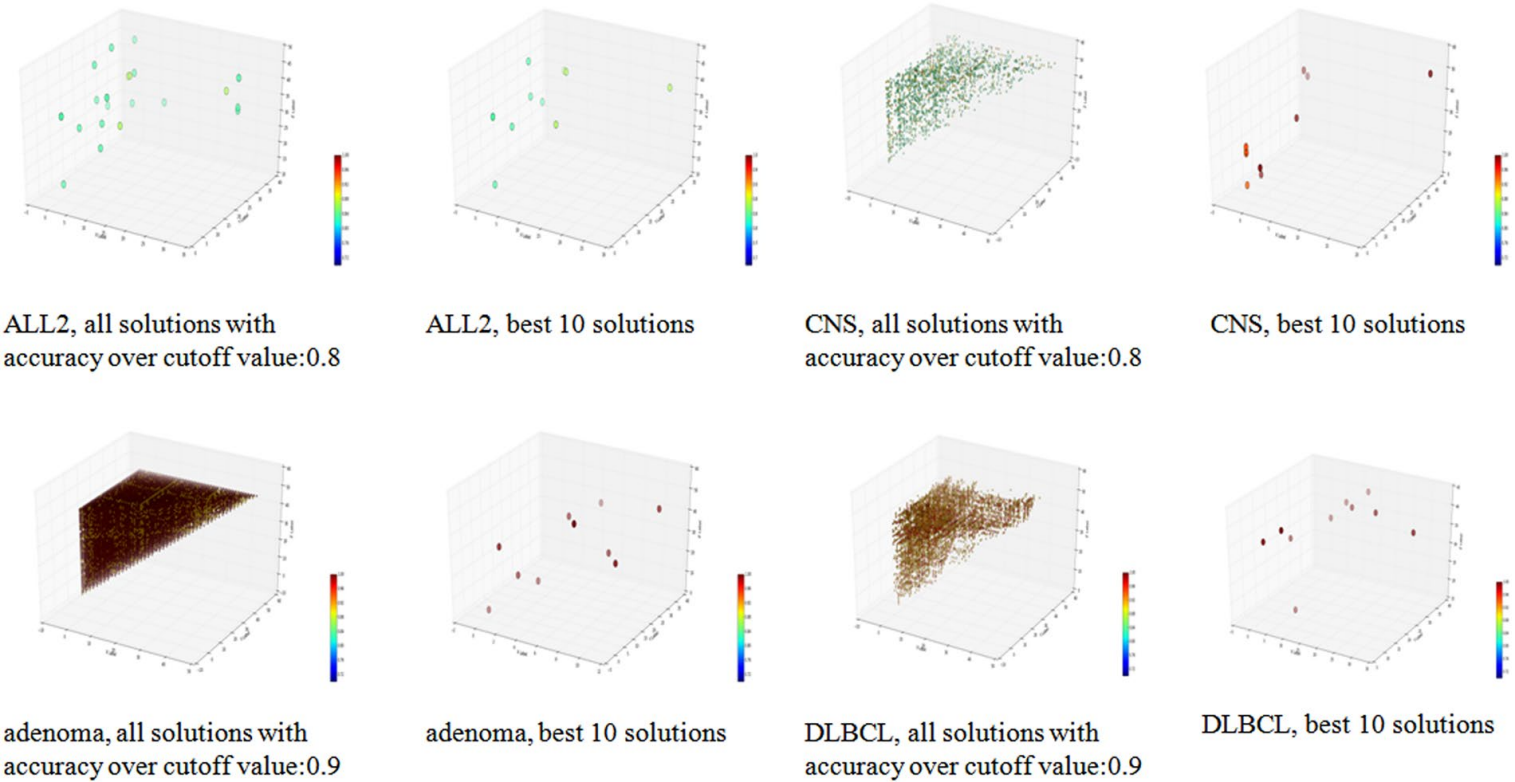
Three-D dot plots for the four datasets. The axises x, y and z are the ranks of each triplet, and x ≤ y ≤ z. The color of each point reflects the accuracy of the model it represents on the corresponding datasets.

Multiple good solutions were detected for all the four datasets, and they scattered across the 3-D space, since x ≤ y ≤ z. The scattering pattern was also observed for the best ten models, as shown in Fig. 2.

Figure 2 shows the scattering map of the selected feature triplets in the 3D plots in Fig. 2. The x, y, z coordinates of a triplet were the ranks of the three features in the top-50 p-value features of t-test. A red color in the heatmap indicates the accuracy of 1.00 and the blue indicates the accuracy of 0.72. For each dataset, the best 10 solutions and the solutions which reach the cutoff value are both shown in the 3D scattering map. For dataset CNS, the best 10 solutions have similar red colors, so they achieved similar classification accuracies. However, they scattered across the 3D space, and clearly demonstrated their differences on the feature compositions. Even more variations were observed for all the solutions with accuracy ≥ 0.8. Similar patterns were also detected for all the other three datasets.

We further investigated whether a good triplet may consist of three features with low classification performances alone. One of the interesting examples is the triplet {D50683_at, HG961-HT961_at, Z35402_rna1_s_at} for the dataset DLBCL. This triplet achieved 95.8% in accuracy, but their individual performances were not very high. The feature D50683_at only achieved 66.7% in accuracy, while the other two features HG961-HT961_at and Z35402_rna1_s_at alone performed slightly better, with accuracies 83.3% and 75.0%, respectively. So it’s necessary to use more features for much better classification performances, and these three features may contribute complementary information for the classification modeling.

### Validations of the above two observations

**Figure 3:**
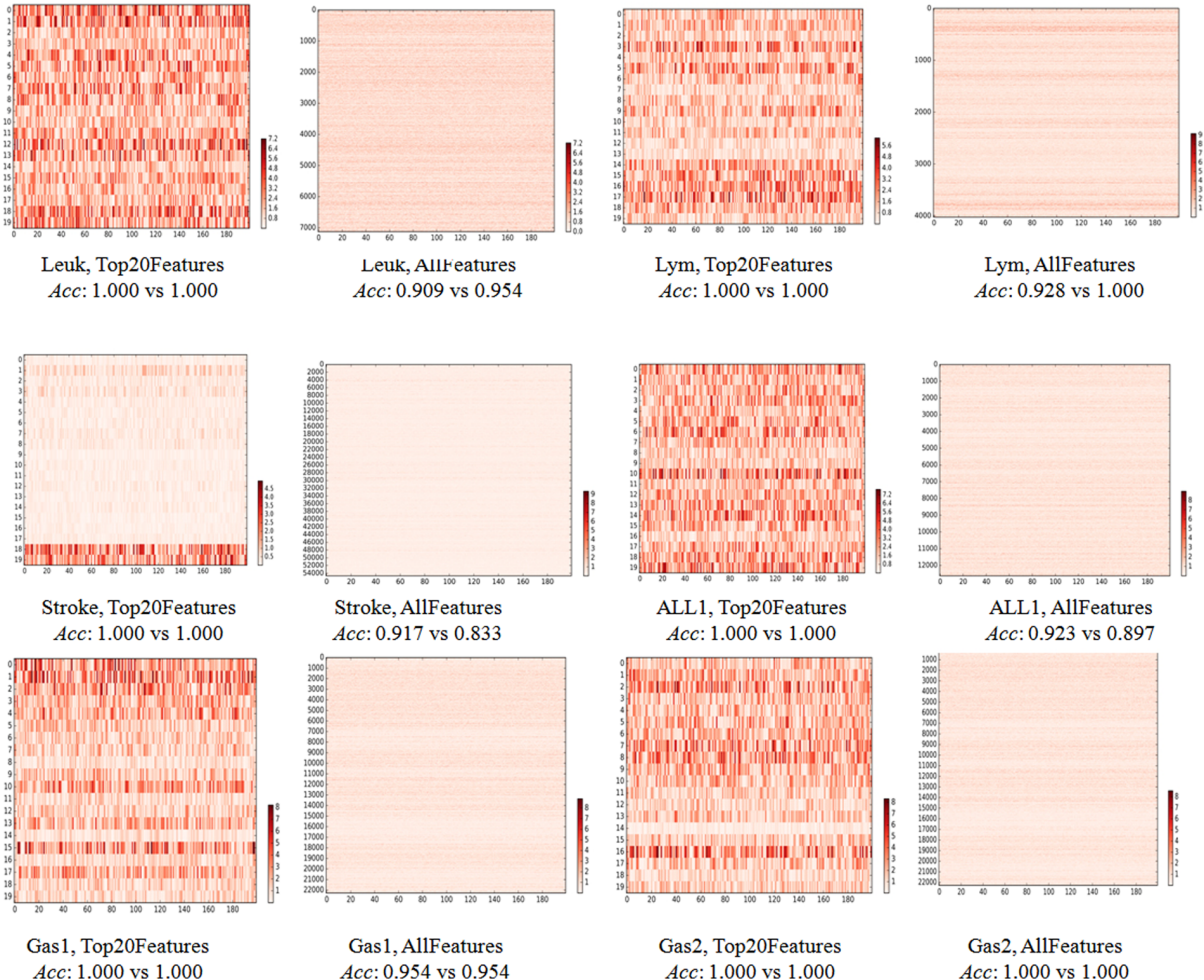
A heatmap of the difference matrix between the best two ELM models for the six independent datasets. If more than two ELM models achieve the best classification accuracy, two models were randomly chosen from these best ones.

The above two observations were further validated on six independent binary classification datasets. The six datasets were described in the section Material and Methods, and the same experimental procedure was carried out on these datasets. The similar significant difference was detected between the best two ELM classification models of each of the six dataset, as shown in Fig. 3 and summarized in Table 3. All the six datasets have at least two ELM models with identical classification performances using the top 20 features ranked by t-test, and these two models were significantly different to each other, as demonstrated by the heatmaps in Fig. 3. Only two of the six datasets Gas1 and Gas2 have the best two ELM models with the same classification accuracies, and they are significantly different to each other, too. The classification accuracies of the best two ELM models of the other cases using all the features in Fig. 3 demonstrated slight differences, which may be due to that only 10,000 random runs of ELM optimizations were conducted in this study. The data also suggested that there exist many noisy features in the biomedical datasets and it’s necessary to remove some before the training of a classification model. In either situation, there holds the existence of multiple similarly-well solutions for the investigated biomedical classification problems.

**Table 3:** Summary of the best ELM models trained on the six independent datasets.

Dataset	Leuk	Lym	Stroke	ALL1	Gas1	Gas2
ELMs in *Top20Features*	5408	4510	653	7861	5477	6545
ELMs in *AllFeatures*	66	31	18	41	145	507
Top20Features: MinAcc(ELM)	0.818	0.867	0.833	0.821	0.818	0.815
Top20Features: MaxAcc(ELM)	1.000	1.000	1.000	1.000	1.000	1.000
AllFeatures: MinAcc(ELM)	0.818	0.857	0.833	0.923	0.818	0.816
AllFeatures: MaxAcc(ELM)	0.954	1.000	0.917	0.821	0.955	1.000

The meaning of each row is similar to Table 1.

**Figure 4:**
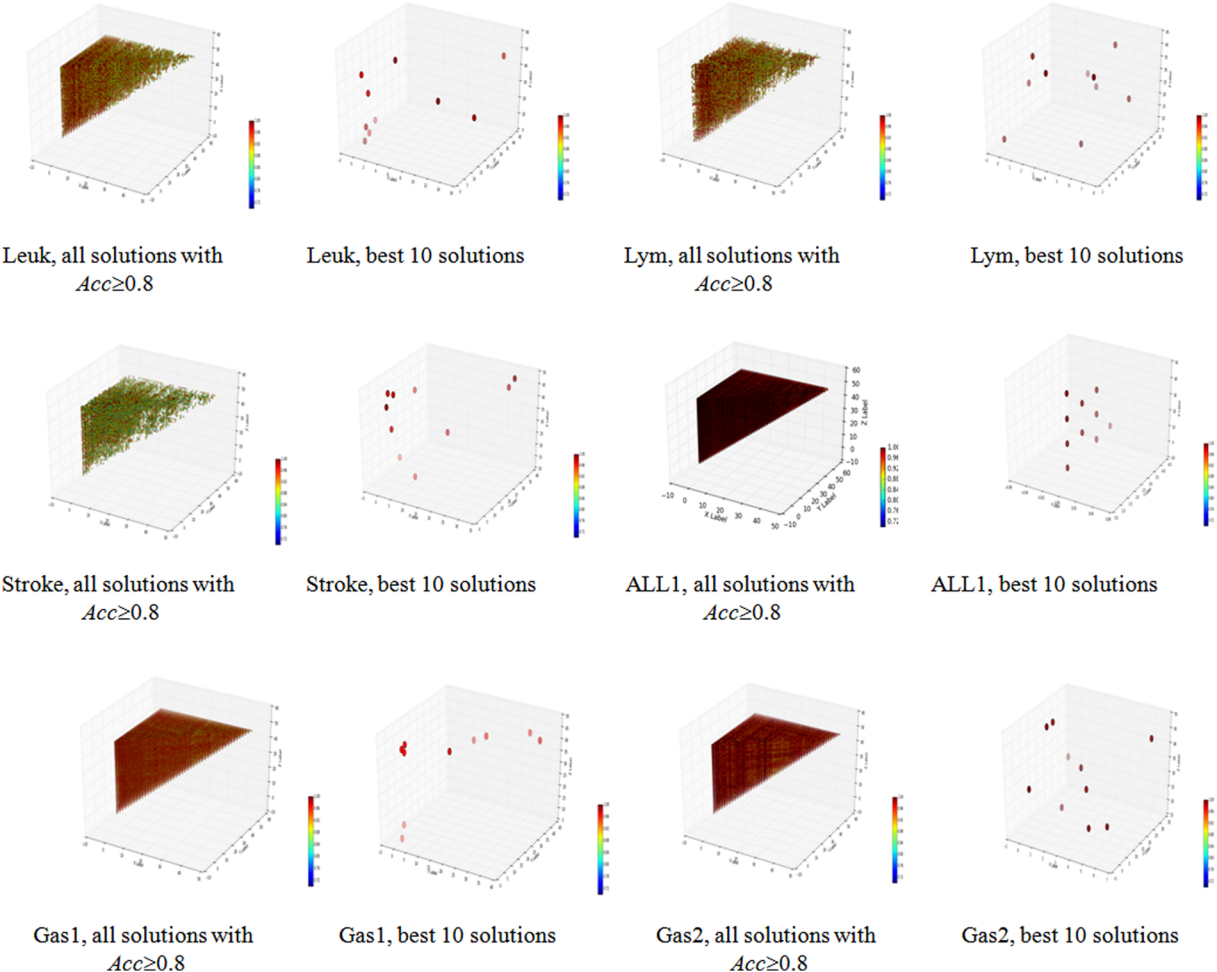
Three-D dot plots for the triplet feature subsets of the six independent datasets using accuracy as the measurement. The axises x, y and z are the t-test ranks of three features in each solution, and x ≤ y ≤ z. The color of each point reflects the accuracy of the model it represents on the corresponding dataset.

All the triplet feature subsets with *Acc* ≥ 0.8 for the six independent datasets were summarized in Fig. 4 and Table 4. We may observe that there are a large number of triplets with *Acc* ≥ 0.8 for each of the six datasets. The averaged rankings of the three features in the best 10 triplets were summarized in the last row of Table 4, and suggested that these features are different to each other. So the best 10 triplets used different features, but they achieved the same classification accuracies, as illustrated in Fig. 4. So there exist multiple feature selection solutions with similarly-well performances for the six independent datasets.

**Table 4:** Summary of the best ELM models trained on the triplets of the six independent datasets.

Dataset	Leuk	Lym	Stroke	ALL1	Gastric1	Gastric2
Triplets	14703	13416	8090	19600	19600	19600
MinAcc(ELM)	1.000	1.000	1.000	1.000	0.980	1.000
MaxAcc(ELM)	1.000	1.000	1.000	1.000	0.980	1.000
(Min, Max)	3.667	4.333	10.667	11.000	23.333	10.333
ranks	17.667	11.667	12.667	6.000	23.667	19.667

The meaning of each row is similar to Table 2.

Like the 4 datasets argued above, the six more datasets investigated in this section have the same situation that features may be complementary to each other and their combination achieved much better. For example, the combination of the three features 208600_s_at, 202469_s_at and 212481_s_at achieved 93.2% in accuracy while they alone can only achieve 79.7% (208600_s_at), 70.4% (202469_s_at) and 86.3% (212481_s_at) in accuracies, respectively.

### There exist multiple feature selection solutions with the best classification precisions or recalls

**Figure 5:**
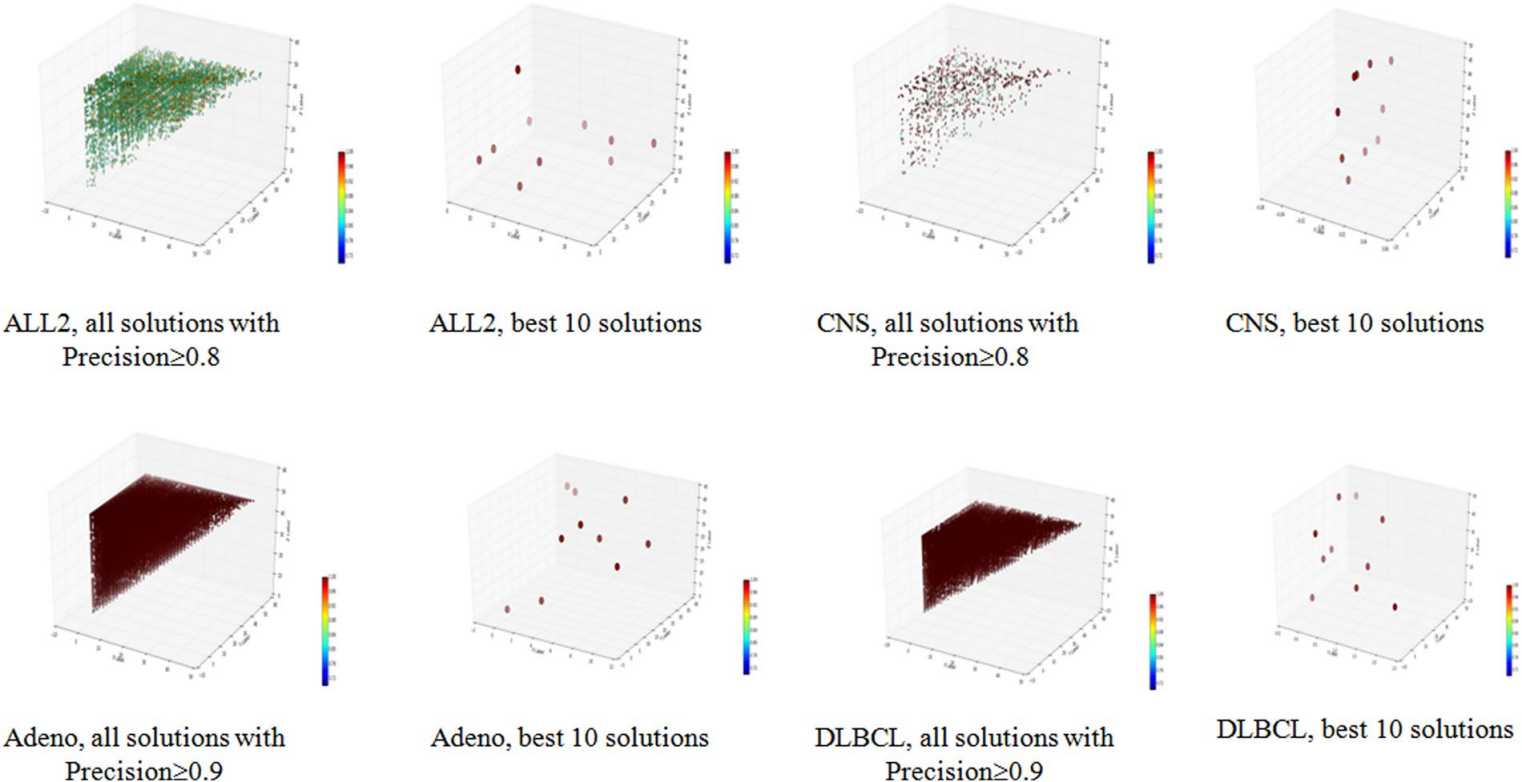
Three-D dot plots for the four datasets using precision as the measurement. The axises x, y and z are the ranks of each solution, and x ≤ y ≤ z. The color of each point reflects the precision of the model on the corresponding datasets.

**Figure 6:**
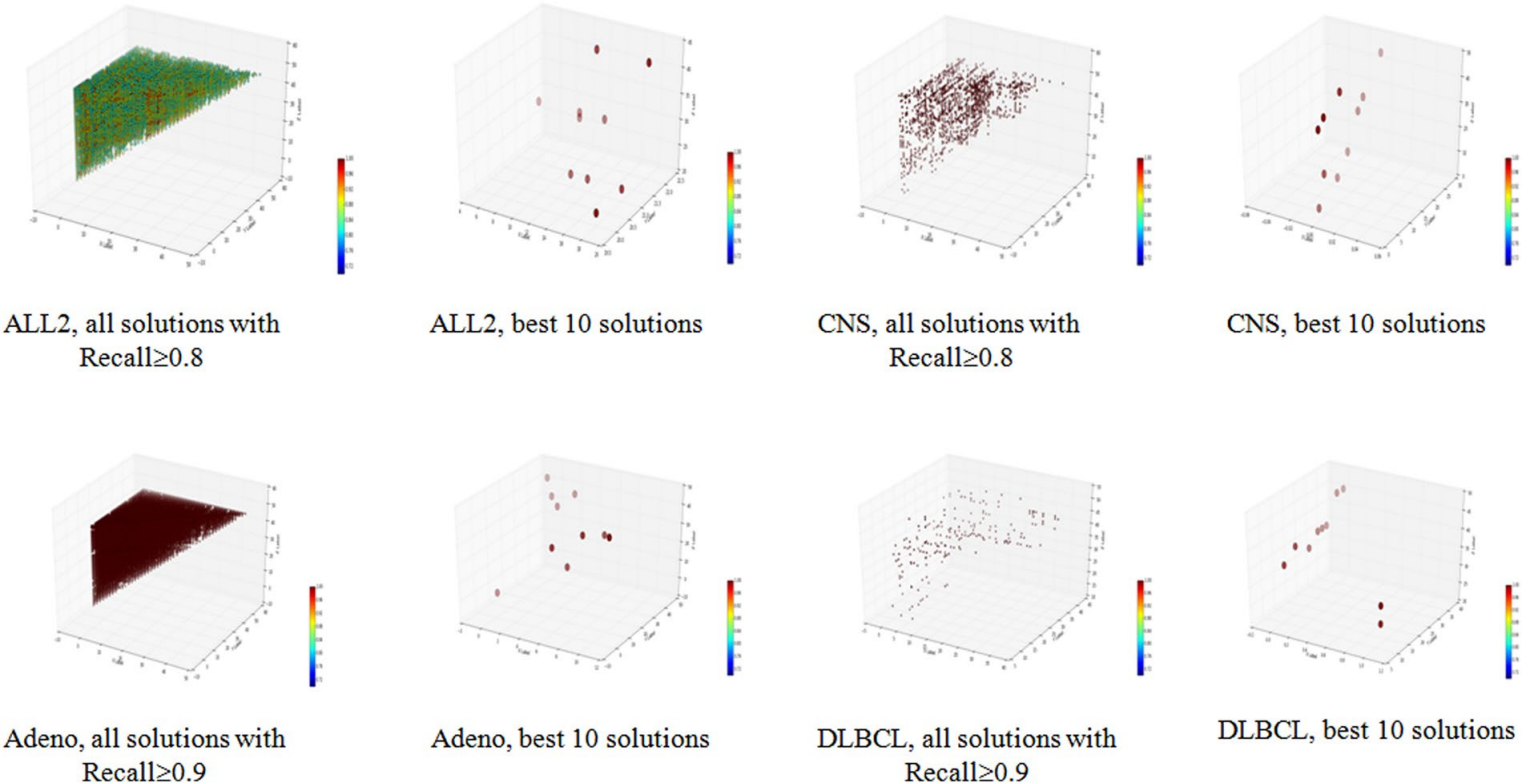
Three-D dot plots for the four datasets using recall as the measurement. The axises x, y and z are the ranks of each solution, and x ≤ y ≤ z. The color of each point reflects the recall of the it represents on the corresponding datasets.

Besides the performance measurement accuracy, precision and recall were also used to evaluate the trained models. Precision was defined as TP/(TP+FP) and recall was defined as TP/(TP+FN), where TP, FP and FN were the numbers of true positives, false positives and false negatives, as defined in^9^. Similar patterns were observed using the two performance measurements precision and recall, as summarized in Table 5 and demonstrated in Figs 5 and 6.

**Table 5:** Summary of the ELM models with the best performance measurements precision and recall trained on triplets of the four datasets.

Dataset	ALL2	CNS	Adeno	DLBCL
Precision(Triplets)	16535	732	17169	14625
MinPrecision(ELM)	1.000	1.000	1.000	1.000
MaxPrecision(ELM)	1.000	1.000	1.000	1.000
(Min, Max) ranks of best-precision 10 models	18.667	10.667	1.333	11.667
	20.333	15.333	24.000	18.667
Recall(Triplets)	4906	1369	17613	198
MinRecall(ELM)	1.000	1.000	1.000	1.000
MaxRecall(ELM)	1.000	1.000	1.000	1.000
(Min, Max) ranks of best-Recall 10 models	19.333	2.000	25.000	19.667
	24.667	4.000	28.333	26.333

The numbers of models with precisions and recalls larger than CutOff were collected for the four datasets ALL2/CNS/Adeno/DLBCL in the row “Precision(Triplets)” and “Recall(Triplets)”, respectively. CutOff is 0.800 for the two difficult datasets ALL2 and CNS, and 0.900 for the two easy datasets Adeno and DLBCL. The minimum and maximum precisions of the best ten ELM models with the precisions larger than the cutoff were listed in the rows “MinPrecision(ELM)” and “MaxPrecision(ELM)”. And the next row gave the averaged rankings of the 10 triplets with the best precisions. The last three rows were defined similarly for the performance measurements Recall.

All the best ten models achieved the same precision 1.000 for all the four datasets although the difficult dataset CNS has much fewer models than the other three datasets, as in Table 5 and Fig. 5. The topological distributions of the best 10 models do not form a tight cluster for all the four datasets, suggesting that these 10 triplets didn’t use similar features. The data in Table 5 also supported this observation that the averaged rankings of three features in the best 10 triplets are different to each other. For example, the dataset Adeno has a triplet with the averaged ranking 1.333, while another of its best 10 triplets has the averaged ranking 24.000.

Significantly different features were used for the 10 triplets with the best recalls for the four datasets. But the numbers of good solutions varied for different performance measurements, suggesting that the solution optimization procedure heavily depends on the optimization goal.

### Biological hints on multiple solutions with the same best classification performances

As shown in the above experiments, there exist multiple solutions with best classification performances, which were demonstrated in ten different datasets. We explored the biological functions of different triplets with the similarly best classification accuracies on the datasets ALL2, CNS, Adeno and DLBCL.

The dataset ALL2 has two groups of features {1803_at, 32783_at, 39271_at} and {36912_at, 1599_at, 1854_at}, and they achieved 0.867 and 0.833 in accuracies, respectively. It was demonstrated that X-ray irradiation may induce G1-arrest in the MOLT-4 lymphocytic leukemia cells, and this process was usually accompanied by reduced activity of CDK2 but increased activity of CDK1 (1803_at)^19^. Another literature suggested that MYBL2 (1854_at) was over-expressed in some acute myeloid leukemias^20^. So it’s interesting to observe that these two leukemia-associated biomarker genes accompanied by two other features may accurately separate the ALL patients with relapse from those without.

The two triplets {L17131_rna1_at, M73547_at, D78012_at} and {U50136_rna1_at, J02611_at, D17793_at} generated 0.889 and 0833 in accuracies for the binary classification problem CNS. The POLYPOSIS LOCUS PROTEIN 1 (M73547_at) has a known association with CNS and patients with familial polyposis have the increased risk of tumor development in extracolonic sites, including the central nervous system^21^. It was also proposed that the expression level of Apolipoprotein D (J02611_at) is correlated with the prognosis in several types of malignancy, including CNS astrocytomas and medulloblastomas^22^.

The dataset Adeno has two triplets {M77836, J02854, T64297} and {H06524, H43887, U37019} among the ten best solutions. These two triplets have not shared features, but both achieved the best classification accuracies 1.000. M77836, also known as pyrroline-5-carboxylate reductase 1 (PYCR1) was experimentally observed as an upregulated protein in an *in vitro* progression model of the colorectal adenoma-to-carcinoma sequence using quantitative 2-DE and is complemented by Western blot validation^23^. Another immunohistochemisty study confirmed that H06524 (Gelsolin precursor) is severely down-regulated in all adenocarcinomas tested while is expressed in normal cells of the colon^24^. So both triplets have genes biologically associated with adenocarcinoma. The existence of these two triplets with the best accuracy 1.000 may represent two distinctive functional modules that could discriminate the adenocarcinoma samples from the normal controls.

The easy dataset DLBCL has two groups of features {X62078_at, L33842_rna1_at, J02645_at} and {X56494_at, M57710_at, U19495_s_at}, both of which achieved 0.917 in accuracy. The gene Lectin, galactoside-binding, soluble, 3 (LGALS3, galectin 3, M57710_at) is a significantly upregulated gene in both the FL and DLBCL lymphoma samples when compared with the expression of the immune escape genes at the single gene level in control DLBCL biopsies using the Oncomine resource^25^. The gene type II inosine monophosphate dehydrogenase (IMPDH2, L33842_rna1_at) is over-expressed in the REF/REL group of DLBCL patients and was suggested to be associated with the resistance to immunochemotherapy of the DLBCL patients^26^. IMPDH2 is also shown to be a negative prognostic factor in other malignancies.

## Conclusion

Our experimental data demonstrated that more than one best solution exist for the disease classification and feature selection problems. Some essential information may be lost if we only focus on one solution for these two optimization problems. So we recommend that at least multiple solutions with very close optimization performances should be delivered by optimization algorithms like classification and feature selection.

## Material and Methods

The python source code for testing ELM, SVM and NBayes on the two difficult datasets ALL2 and CNS may be freely accessed at http://www.healthinformaticslab.org/supp/.

### Proposed Methodology of Disease Classification

Disease diagnosis is a binary classification problem that we determine whether a given sample has the investigated disease or not based on its data. There are two groups of samples in such a problem. There is usually one group of samples carrying a specific disease, and the other group of samples consists of the control samples. A highly accurate classification model may greatly help the clinical doctors improve the diagnosis sensitivity and reduce the false positives.

ELM (Extreme Learning Machine) is a machine learning algorithm that implements a feed-forward neural network with a single layer of hidden nodes for classification or regression. ELM features generating a very good classification model even with the utilization of a vector of randomly generated input weights. It’s also very efficient on dealing with a huge amount of features.

So we hypothesize that there may be multiple ELM classification models with similar prediction accuracies. The low computational requirement of ELM makes it possible to run ELM multiple times within a reasonable period of time.

### Experimental settings of Disease Classification

We chose four publicly available datasets, *i.e*. ALL2, CNS, Adeno and DLBCL. These four datasets are representative since the datasets ALL2 and CNS are difficult in classification while the other two datasets Adeno and DLBCL are easy to be separated, as shown in a recent work^9^. All these datasets are binary classification problems.

Two experimental strategies were employed to find multiple optimized ELM models. Firstly, features in each dataset were evaluated for their discriminative abilities using t-test, and only 20 features with the smallest P-values were kept for ELM constructions. This round of experiments was denoted as *Top20Features*. Secondly, all the features in a dataset were used to train the ELM models, and this round is denoted as *AllFeatures*.

10,000 optimizations of ELM models using different randomly generated input weights were carried out for each round of experiments. Each dataset was split into two parts, i.e. 70% as the training and 30% as the test datasets. The binary classification accuracy on the test dataset was calculated for comparison, as defined in^9^.

The ELM module Python-ELM version 0.3 of the Python programming language was used in this study^27^. This module implements an MLP (Multi-Layer Perceptron) for the random input layer. For a dataset with *m* features, an *n* × *m* weight matrix will be generated based on the training data, where *n* is the number of units in the layer, and *n* are 150 and 290 for the modes Top20Features and AllFeatures, respectively. This matrix was extracted to represent the trained ELM model, and a comparison was conducted between the weight matrices of two ELM models. A difference matrix of two weight matrices is defined to be the absolute value of the first matrix minus the second one. All the experiments in this study were carried out in an Inspur Gene Server G100, with 256GB memory, 28 Intel Xeon® CPU cores (2.4 GHz), and 30TB RISC1 disk space.

### Proposed Methodology of Feature Selection

Besides the classification problem, we also investigated the existence of multiple similarly good feature subsets for the four datasets, *i.e*. ALL2, CNS, Adeno and DLBCL. Almost all the existing feature selection algorithms are deterministic, and they generate only one solution. So we chose the top 50 ranked features based on the t-test Pvalues for the four datasets, and conducted an exhaustive screening of all the triplets from these 50 features. Each dataset is randomly split into two parts: 80% as training dataset and 20% as test dataset. The accuracy of test dataset is used to represent the binary classification accuracy. The accuracy cutoff 0.8 was chosen for the two difficult datasets ALL2 and CNS, and 0.9 for the two easy datasets Adeno and DLBCL. Besides accuracy, two other performance measurements precision and recall were used to test our hypothesis. The same cutoff values were used to screen the solutions, so that the solutions with top performances were highlighted.

### An extended validation on six more datasets

An extended validation on six more binary-classification transcriptomic datasets were employed to further support our hypothesis. The widely used Leukaemia (Leuk) dataset has 47 acute lymphoblastic leukaemia (ALL) and 25 acute myeloid leukaemia (AML) samples^28^. Another dataset Lymphoma (Lym) has 22 germinalcentre and 23 activated B-like DLBCL samples^29^. The 40 samples of the dataset Stroke (accession: GSE22255) consists of 20 ischemic stroke patients and 20 controls^30^. One more dataset ALL1 was chosen from the same database of ALL2, and it has 95 B-cell and 33 T-cell acute lymphoblastic leukaemia (ALL) samples^31^. The two gastric cancer datasets Gas1 and Gas2 were retrieved from the database Gene Expression Omnibus Gastric1/Gastric2 (accession: GSE29272)^32^. Gas1 consists of 72 non-cardia gastric cancer samples and 72 normal controls. Gas2 has 62 cardia gastric cancer samples and 62 normal controls.
