# Author Correction: Multiple similarly effective solutions exist for biomedical feature selection and classification problems

**DOI:** 10.1038/s41598-018-19383-1

**Published:** 2018-01-11

**Authors:** Jiamei Liu, Cheng Xu, Weifeng Yang, Yayun Shu, Weiwei Zheng, Fengfeng Zhou

**Affiliations:** 1 College of Software, Jilin University, Changchun, Jilin 130012 China; 2 College of Computer Science and Technology, and Key Laboratory of Symbolic Computation and Knowledge Engineering of Ministry of Education, Jilin University, Changchun, Jilin 130012 China

Correction to: *Scientific Reports* 10.1038/s41598-017-13184-8, published online 09 October 2017

The original version of this Article contained typographical errors in the title, where:

“Multiple similarly-well solutions exist for biomedical feature selection and classification problems”

now reads:

“Multiple similarly effective solutions exist for biomedical feature selection and classification problems”

This has now been corrected in the HTML and PDF versions of this Article.

