# Publisher Correction: Multiple similarly effective solutions exist for biomedical feature selection and classification problems

**DOI:** 10.1038/s41598-018-22352-3

**Published:** 2018-03-06

**Authors:** Jiamei Liu, Cheng Xu, Weifeng Yang, Yayun Shu, Weiwei Zheng, Fengfeng Zhou

**Affiliations:** 1 College of Software, Jilin University, Changchun, Jilin 130012 China; 2 College of Computer Science and Technology, and Key Laboratory of Symbolic Computation and Knowledge Engineering of Ministry of Education, Jilin University, Changchun, Jilin 130012 China

Correction to: *Scientific Reports* 10.1038/s41598-017-13184-8, published online 09 October 2017

An error occurred after publication resulting in the inadvertent omission of the Article and Volume numbers from the PDF version of this Article. This error has now been corrected in the PDF version of the Article; the HTML version was correct from the time of publication.

